# Synthetic computed tomography for low-field magnetic resonance-only radiotherapy in head-and-neck cancer using residual vision transformers

**DOI:** 10.1016/j.phro.2023.100471

**Published:** 2023-07-08

**Authors:** Agustina La Greca Saint-Esteven, Ricardo Dal Bello, Mariia Lapaeva, Lisa Fankhauser, Bertrand Pouymayou, Ender Konukoglu, Nicolaus Andratschke, Panagiotis Balermpas, Matthias Guckenberger, Stephanie Tanadini-Lang

**Affiliations:** a Department of Radiation Oncology, University Hospital Zurich and University of Zurich, Rämistrasse 100, Zurich 8091, Switzerland; b Computer Vision Laboratory, Department of Information Technology and Electrical Engineering, ETH Zurich, Sternwartstrasse 7, Zurich 8092, Switzerland

## Abstract

**Background and purpose:**

Synthetic computed tomography (sCT) scans are necessary for dose calculation in magnetic resonance (MR)-only radiotherapy. While deep learning (DL) has shown remarkable performance in generating sCT scans from MR images, research has predominantly focused on high-field MR images. This study presents the first implementation of a DL model for sCT generation in head-and-neck (HN) cancer using low-field MR images. Specifically, the use of vision transformers (ViTs) was explored.

**Materials and methods:**

The dataset consisted of 31 patients, resulting in 196 pairs of deformably-registered computed tomography (dCT) and MR scans. The latter were obtained using a balanced steady-state precession sequence on a 0.35T scanner. Residual ViTs were trained on 2D axial, sagittal, and coronal slices, respectively, and the final sCTs were generated by averaging the models’ outputs. Different image similarity metrics, dose volume histogram (DVH) deviations, and gamma analyses were computed on the test set (n = 6). The overlap between auto-contours on sCT scans and manual contours on MR images was evaluated for different organs-at-risk using the Dice score.

**Results:**

The median [range] value of the test mean absolute error was 57 [37–74] HU. DVH deviations were below 1% for all structures. The median gamma passing rates exceeded 94% in the 2%/2mm analysis (threshold = 90%). The median Dice scores were above 0.7 for all organs-at-risk.

**Conclusions:**

The clinical applicability of DL-based sCT generation from low-field MR images in HN cancer was proved. High sCT-dCT similarity and dose metric accuracy were achieved, and sCT suitability for organs-at-risk auto-delineation was shown.

## 1 Introduction

Recent technological advances have facilitated the integration of a magnetic resonance (MR) imaging unit with a linear accelerator (MR-Linac), enabling MR-guided radiation therapy (MRgRT) [1]. Depending on the available commercial system, the MR scanner may be low- or high-field. The latter can acquire higher-quality images but is significantly more expensive and more susceptible to artefacts and geometric distortions than the former [2]. Patient imaging before and during dose delivery allows for plan adaptation and beam gating which, together with the improved soft tissue definition, make MRgRT a more personalised choice in cancer treatment [3], [4]. Moreover, there is increasing evidence addressing its possible benefits for treating head-and-neck (HN) cancer patients [5], [6].

MRgRT requires, however, the acquisition of a computed tomography (CT) image to perform dose calculation on the simulation day. In the following treatment days, deformable image registration (DIR) of the original CT to the new MR scan is performed, or a combination of DIR and bulk density override [7]. This method not only increases patient radiation exposure, but also introduces uncertainties, estimated to be around 2 mm [8], [9]. In a complete MR-only radiotherapy workflow, CT acquisition should be replaced with an accurate synthetic CT (sCT) generation method. Different approaches have been proposed (and some commercially implemented [10], [11]) for multiple cancer sites which have brought MR-only radiotherapy closer to its clinical implementation [8], [12], [13], [14]. Among these, deep learning (DL) methods have shown great potential with little compromise on time requirements, operator dependencies, and registration errors, as opposed to atlas-based and bulk override methods [15], [16], [17]. Dose deviations between CT and DL-generated sCT scans are typically lower than 2% [12], fulfilling the requirement for clinical applicability [18].

Since the first application of a convolutional neural network (CNN) for MR-to-CT image synthesis in 2016 [19], the scope of DL architectures and training strategies for this task has increased at a vertiginous pace [12], [13]. CNNs trained with pixel-wise losses have been outperformed by generative adversarial networks (GANs) [20], which employ adversarial losses to learn the target distribution conditioned on the source modality. GANs are composed of one or two generator/discriminator pairs, which compete to generate/discriminate realistic fake images. However, GANs also present some drawbacks, such as training instability, which can result in vanishing gradients or mode collapse [21], together with limited learning of global spatial dependencies and inter-subject generalisation [22]. More recently, vision transformers (ViTs) [23] have achieved state-of-the-art performance in a wide range of medical image analysis tasks, including sCT generation [24]. Transformers incorporate self-attention mechanisms that allow them to obtain contextual representations and improve the detection of long-range dependencies. The combination of ViTs and CNNs has also been explored recently, which has not only enabled the joint learning of local and global details but has also alleviated the computational burden of ViTs [24]. In the field of medical image synthesis, hybrid architectures have been proposed which involve the integration of transformer blocks within the generator and/or discriminator networks of convolutional GANs, such as GAN-BERT [25], VTGAN [26], SLATER [27], and residual vision transformers (ResViT) [28].

Specific to the HN region, ten previous studies employed DL to generate sCT scans from MR images [29], [30], [31], [32], [33], [34], [35], [36], [37], [38]. These MR scans were acquired using high-field scanners and T1 or T2 sequences, except for two studies that utilised multi-parametric series. The architectures employed were deep CNNs and GANs, predominantly trained on paired 2D axial slices. Reported results showed a mean absolute error (MAE) ranging from 65 to 131 Hounsfield units (HU) and dose-volume histogram (DVH) deviations between 0.5 and 6%. Nevertheless, only two studies incorporated multi-view slices and none of them explored the use of low-field MR scans. The latter has only been investigated for abdomen, prostate, and lung cancer [39], [40], [41], [42], [43], [44], [45], resulting in promising image similarity scores (MAE = 26–90 HU) and dose metric accuracies (DVH = 1–5%). However, and, like the HN cancer studies, none of these studies investigated the use of ViTs.

Here we present the first study on the generation of sCT scans from low-field MR images in the HN region to advance MR-only radiotherapy. Moreover, unlike similar previous literature, the application of the hybrid model ResViT was explored, as well as the use of orthogonal sets of 2D slices to incorporate 3D information. To evaluate the clinical feasibility of the proposed method, an analysis of the dose metric accuracy and four global gamma analyses were carried out. Additionally, the suitability of sCT scans for organs-at-risk (OARs) auto-delineation was assessed to determine the similarity to manual contours and whether this step could be integrated into the treatment planning workflow.

## 2 Materials and methods

### 2.1 Study cohort

Imaging and clinical data from 31 HN cancer patients treated with MRgRT at the University Hospital of Zurich between August 2020-December 2022 were retrospectively collected. Patients gave prior informed consent, and the use of the data was approved by the local ethics committee (2018–01794 and 2019–00993). A detailed description of the treatment can be found in [5], [6]. For most patients, one MR-CT pair from the simulation day was available, together with 5–6 MR scans from the treatment days. Because the training-validation-test split was performed with no patient overlap among cohorts, to maximise the amount of training data, patients with a lower number of available images were assigned to the test set. The remaining patients were assigned randomly to one cohort following these proportions: training ∼65% (n = 20), validation ∼15% (n = 5), and test ∼20% (n = 6). A flow diagram describing the study design can be found in Supplement A. The patients’ characteristics are summarised in Supplement B.

### 2.2 Image acquisition

Mixed T1/T2-weighted MR images were acquired using the 0.35T scanner of the MRIdian system (ViewRay, Ohio, USA) with a balanced steady-state precession sequence and a dedicated HN coil. A custom 5-point thermoplastic mask and a cushion (CIVCO Radiotherapy, Iowa, USA) were employed for patient immobilisation. The cubic resolution was set to 1.5×1.5×1.5 mm^3^ and the matrix size was 202×360. On the simulation day, a CT scan (Somatom Definition AS, Siemens, Erlangen, Germany) was also acquired within 120 min of the MR acquisition with the same immobilisation setup. The in-plane resolution ranged from 0.98 to 1.56 mm, whereas the axial resolution was 2 mm for all scans. The matrix size was set to 512×512. Despite minimal, the differences were handled via DIR in the MRIdian treatment planning system using the default settings as described in [5]. The resulting deformed CT (dCT) scans had the same in-plane and axial resolution as the MR scan. For each treatment fraction, the original CT was registered to the new MR scan following the same procedure. The radiotherapy dose plan and structure set with the planning target volume (PTV), gross tumour volume (GTV), and OARs were available for each treatment day. The delineations were carried out by an experienced radiation oncologist, who also reviewed and approved the MR-dCT registrations.

### 2.3 Image pre-processing

Three different sets of 2D slices, one for each orthogonal direction, were collected. For each set, the slices included spanned the PTV with a 20 mm margin in the respective directions (i.e., craniocaudal for the axial set, mediolateral for the sagittal set, and anteroposterior for the coronal set), as shown in Supplement C. The CT slices which contained artefacts caused by dental implants or the contrast agent were excluded, as well as their corresponding paired MR slice. This was done to prevent the model from erroneously creating artefacts. The number of remaining slices included in the study can be found in Supplement A. These slices were cropped around their respective centre to have a shape of 256×256 pixels. CT scans were clipped in the range of −1024 HU to 1200 HU and min-max normalised. MR scans were min-max normalised per-patient after clipping the intensities between the 2*th*- and 98*th*-percentiles values. Each pair of MR-dCT slices was masked with a binary body mask to eliminate potential confounding artefacts, such as the table and the thermoplastic mask. A 3D body mask was also constructed via the union of the axial, sagittal, and coronal body masks (Supplement C).

### 2.4 Network: Architecture and training

The model ResViT was employed, which was made publicly available by Dalmaz et al. [28]. ResViT is similar to classical GANs as it is also composed of one generator and one discriminator that compete against each other. However, the novelty relies on the inclusion of the so-called aggregated residual transformer blocks (ART) in the generator, which together with deep convolutional operators and the residual connections between them, enable the incorporation of local and contextual details. The conditional discriminator, on the other hand, is based solely on convolutional operators. As a result, ResViT captures long-range contextual information, while preserving local details through convolutions and realism through adversarial training.

As recommended by the authors, the network was first trained without transformers for 50 epochs. Then, the ART blocks, pre-trained on the ImageNet dataset, were inserted and the model was trained for another 50 epochs. The default hyper-parameter values specified by Dalmaz et al. were used [28]. The loss function minimised via Adam optimizer was a sum of two different terms: the L1 loss computed pixel-wise between the dCT and the sCT weighted by λ_pixel_, and the adversarial loss weighted by λ_adv_ (Equations D1-3 in Supplement D). Data augmentation was performed on each epoch using the library Monai [46] and consisted of random affine transformations with a probability of 0.8 (ranges: ±10◦ for rotation, ±15 pixels for translation, and ±0.1 for shear), as well as random left-right flips with a probability of 0.5. The transformations were applied to each pair of MR-dCT slices. For each orthogonal view, the final model was selected based on the epoch with the lowest MAE on the validation set.

### 2.5 Image post-processing

**Fig. 1:**
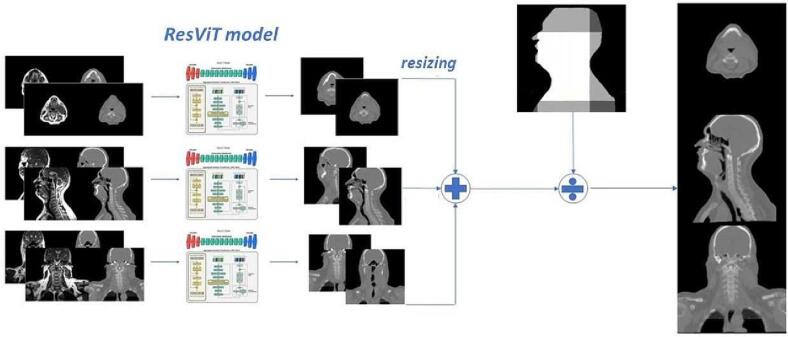
Three models are trained on axial (top), sagittal (middle), and coronal (bottom) 2D slices, respectively. The outputs are averaged using a 3D body mask. ResVit model image taken from [28].

The 256×256 axial, sagittal, and coronal sCT 2D slices were respectively concatenated together to obtain three different sCT volumes. These were later resized to the original size of the corresponding dCT and averaged to obtain the final sCT (Fig. 1). The averaging was done using only the voxels >0 in the 3D body mask.

### 2.6 Evaluation

At test time, there was no exclusion of slices with artefacts to allow for a complete comparison of the original dCT-based and the sCT-based dose distributions. However, the voxels with a HU value ≥2200 in the dCT (i.e., voxels with artefacts) were not considered in the computation of the following image similarity metrics (Equations D4-7 in Supplement D): MAE, root mean squared error (RMSE), peak-signal-to-noise ratio (PSNR) and structural similarity index measure (SSIM). For each patient, the different metrics were calculated pixel-wise and averaged within the 3D body mask.

Each test sCT scan was converted to an electron density map, onto which the dCT-based dose plan was rigidly copied and recalculated using Monte Carlo algorithm (grid size of 3 mm, magnetic field corrections activated, variance of 1%). The following points were used to evaluate the DVH differences: the coverage (D_95%_), near- maximum (D_2%_), and mean dose (D_mean_) to the PTV, the D_2%_ to the spinal cord and mandible, and the D_mean_ to the GTV, parotid glands, submandibular glands, and oral cavity. Furthermore, a total of four global 3D gamma analyses were performed using 2%/2mm and 3%/3mm passing criteria and 50% (25 Gy) and 90% (45 Gy) of the prescribed dose as thresholds.

The commercial software solution Contour ProtégéAI™ v1.1.2 (MIM Software Inc., Cleveland, USA) was employed to automatically delineate six different OARs (the left and right parotid glands and submandibular glands, the oral cavity, and the mandible) on the generated sCTs and the dCTs of the test patients. The Dice similarity coefficient (DSC, equation D8 in Supplement D) was employed to assess the segmentation accuracy with respect to the contours manually delineated on the MR images by a radiation oncologist.

## 3 Results

The final axial, sagittal, and coronal models corresponded to epochs 85, 84, 79, respectively. On average, the training process of one model took 87 h, whereas the generation of an sCT volume at test time took less than 2 min per patient. The dose plan recalculation on the sCT took less than 10 min. Detailed results for the six test patients can be found in Supplement E.

### 3.1 Image similarity

**Fig. 2:**
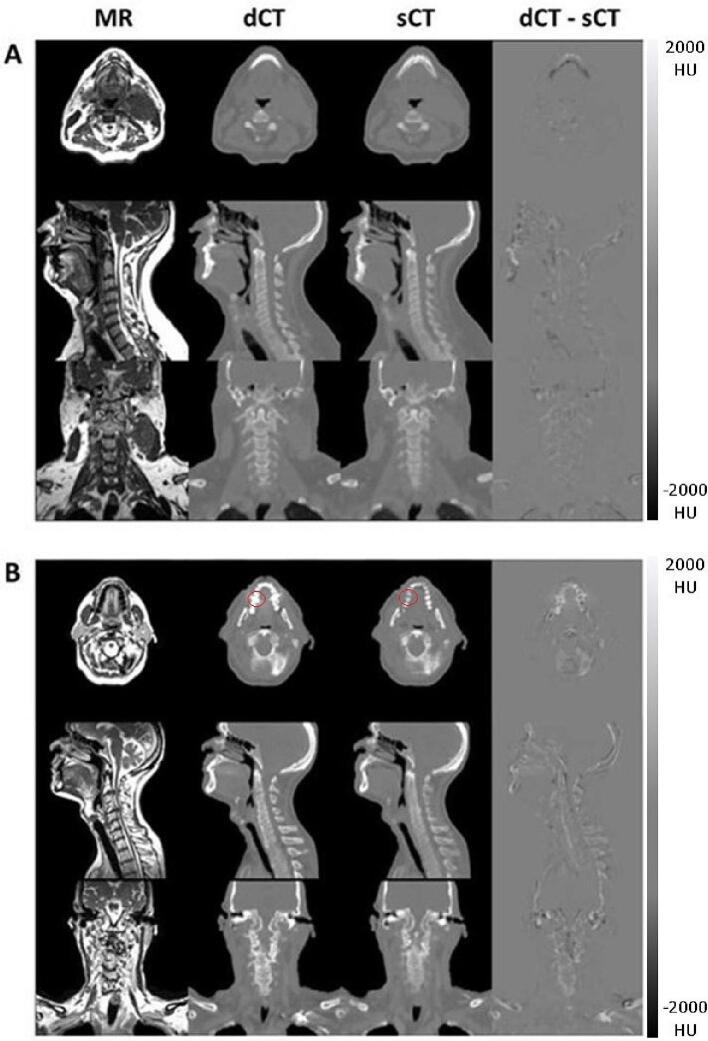
A: Axial, sagittal, and coronal views of the sCT scan which achieved the lowest MAE (37 HU). B: Axial, sagittal, and coronal views of the sCT scan which achieved the highest MAE (74 HU).

On the test set, the achieved metrics (median [range]) were as follows: MAE of 57 [37–74] HU, RMSE of 117 [75–149] HU, PSNR of 0.98 [0.97–0.99], and SSIM of 30.9 [28.8–34.7]. Different MR, dCT, and sCT slices from the test subject with the lowest MAE (37 HU) are shown in Fig. 2A, while the same is depicted for the subject with the highest MAE (74 HU) in Fig. 2B. Similar images for the remaining test patients can be found in Supplements F-G. As one can see in Fig. 2B, the presence of dental artefacts (axial view) and the contrast agent (coronal view) was neglected by the network, which instead assigned lower HU values to the affected voxels.

### 3.2 Dose metric accuracy analysis

**Fig. 3:**
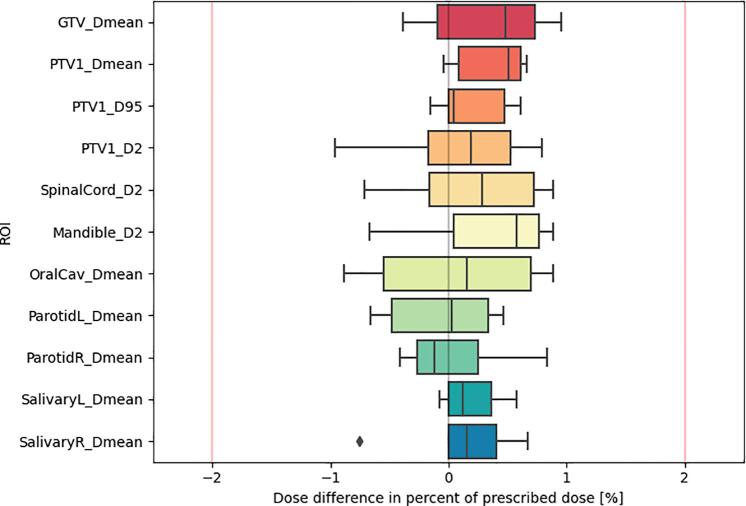
Differences between the sCT-based and the dCT-based DVHs for different points: Spinal D_2%_ to the PTV, spinal cord and mandible; D_mean_ to the left and right submandibular glands, left and right parotid glands, oral cavity, GTV, and PTV.

**Fig. 4:**
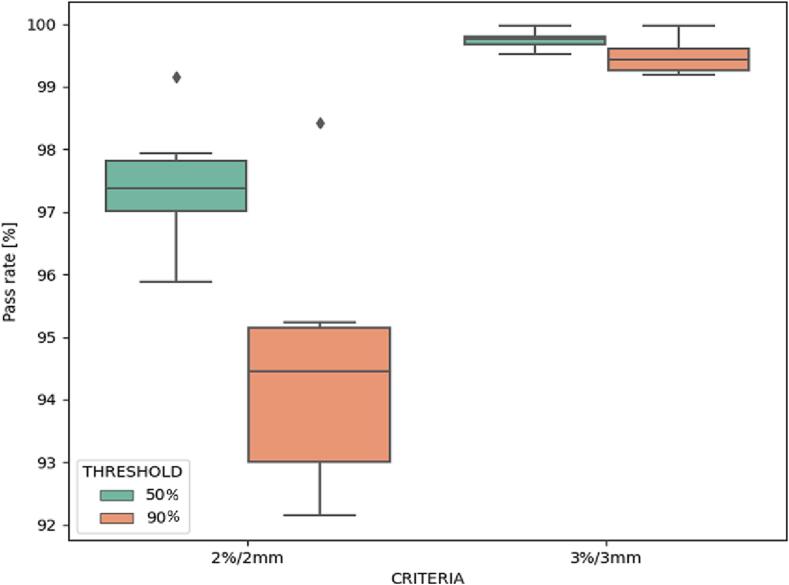
Boxplot of the gamma pass rates using 2%*/*2mm and 3%*/*3mm criteria. The dose thresholds are set to 50% and 90% of the prescribed dose (50 Gy).

The DVHs of the six test patients can be found in Supplement H. The boxplot of the relative signed DVH differences is shown in Fig. 3. No deviations above 1% (0.5 Gy) were found. The median [range] passing rates for the 2%/2mm analyses were 97.4 [96.9–99.2] and 94.6 [94.0–98.4] for the 50% and 90% thresholds respectively, whereas for the 3%/3mm analyses they were 99.8 [99.7–100.0] and 99.5 [99.2–100.0] (Fig. 4).

### 3.3 Auto-delineation accuracy

**Fig. 5:**
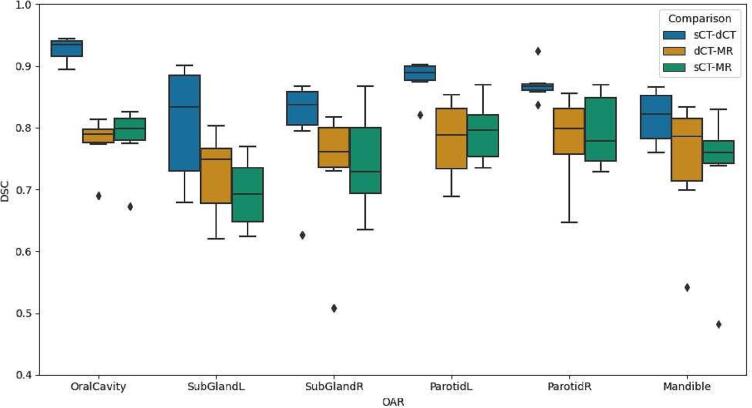
Boxplot of the calculated DSCs between the automatically generated contours on the sCT and the automatically generated contours on the dCT (blue); between the automatically generated contours on the dCT and the manual contours on the MR (orange); and between the automatically generated contours on the sCT and the manual contours on the MR (green). (For interpretation of the references to colour in this figure legend, the reader is referred to the web version of this article.)

Fig. 5 shows the DSCs calculated between the different sets of contours obtained automatically on the sCT and the dCT scans, and manually on the MR images. The highest agreement was observed for the oral cavity and the left parotid gland, whereas the submandibular glands presented the worst results. Similar boxplots can be found in Supplement I for other segmentation metrics.

## 4 Discussion

The proposed multi-view method achieved excellent performance with a final median [range] MAE of 57 [37–74] HU on the internal test set. All DVH deviations were lower than 1% of the prescribed dose and the gamma analyses indicated a high agreement between the original and the sCT-based dose plans. Additionally, the generated sCT scans were proven suitable for OARs auto-contouring using a commercial software solution.

The main challenge encountered in this study was the small dataset size, primarily due to the limited availability of 0.35T MR-Linac machines worldwide. Additionally, considering the uniqueness of the MR sequence employed, it was not feasible to augment the dataset with additional data from other sources, as the mixed T1/T2 contrast obtained is distinct and not directly comparable to T1 or T2-weighted images. To increase the effective dataset size, 2D and deformably registered MR-CT pairs were employed. This registration process served as a form of data augmentation at the expense of potentially including registration errors in the learning process. The need for registered image pairs was dictated by the choice of the model, and could be mitigated with the inclusion of a registration block [47] or the use of cycle-consistency [48] and contrastive [49] losses, at the cost of increased computation time. Another measure could be the adoption of a semi-supervised training strategy, which would allow for the use of unpaired data [50] or under-sampled datasets [51].

The model ResViT was selected because it was shown to outperform different state-of-the-art models in pelvic sCT generation from high-field MR images by its authors [28]. Notably, ResViT exhibited superior results in bony regions, lower artefacts, more accurate soft tissue depictions, and improved inter-subject generalisation. Our approach also achieved the lowest reported MAE when compared to previous literature on MR-to-CT generation in HN cancer (Supplement J). However, due to the differences in the magnetic fields and MR sequences employed, a straightforward comparison with other studies is not possible. Moreover, the differences in the reported similarity metrics, including average calculation within 3D volumes or 2D slices, and specific tissue selection using varying thresholds, posed challenges in comparing the studies. Similarly, three out of the ten studies were lacking a dose accuracy analysis, with the remaining seven presenting substantial variations in the criteria for gamma analyses (2D versus 3D) and thresholds employed, as well as in the points used to assess DVH deviations. When compared to other studies on low-field MR-to-CT synthesis for other cancers, our approach performed comparably well achieving lower than average MAE values and DVH deviations (Supplement J). However, it should be noted again that our dataset size was very limited, and that a direct comparison is not possible due to differences in the metrics computations.

Another limitation present in this study was the prevalence of dental artefacts, which is known to greatly influence the performance of image analysis tools. The affected 2D slices were excluded during training to prevent the model from generating them, but this resulted in a compromised fidelity of the synthetic images, as shown in Fig. 2. Nevertheless, the DVH deviations for the mandible D_2%_ and the oral cavity D_mean_ remained lower than 1%, and the median DSCs between the auto-contours on the sCT and the manual contours on the MR for these structures were 0.76 and 0.80, respectively.

Lastly, the model’s generalisation capability to other cancer sites, other MR-Linac scanners, and other treatments, such as proton therapy, could be further evaluated to prove the clinical utility of the method. Similarly, before this model can be implemented in the clinic, a quality assurance protocol remains to be performed. Once this is accomplished, the method’s performance on a real clinical workflow, as the ones suggested in [5], [52], could be studied, focusing on its quality and time sensitivity, as it currently takes ∼12 min for sCT generation and plan recalculation.

To conclude, this study proved the feasibility of DL-based sCT generation from low-field MR images in the HN region, bringing MR-only radiotherapy closer to its clinical application. The achieved DVH deviations were below 1%, fulfilling the clinical applicability criteria. Moreover, the sCT scans were proven suitable for OARs auto-delineation, ensuring a potential smooth integration in the workflow.

## CRediT authorship contribution statement

**Agustina La Greca Saint-Esteven:** Conceptualization, Methodology, Software, Validation, Formal analysis, Investigation, Data curation, Writing – original draft, Visualization. **Ricardo Dal Bello:** Conceptualization, Methodology, Software, Validation, Formal analysis, Resources, Writing – review & editing. **Mariia Lapaeva:** Software, Writing – review & editing. **Lisa Fankhauser:** Software, Data curation, Writing – review & editing. **Bertrand Pouymayou:** Formal analysis, Validation, Writing – review & editing. **Ender Konukoglu:** Supervision, Resources, Writing – review & editing. **Nicolaus Andratschke:** Conceptualization, Writing – review & editing, Supervision, Project administration, Funding acquisition. **Panagiotis Balermpas:** Data curation, Conceptualization, Writing – review & editing, Supervision, Project administration, Funding acquisition. **Matthias Guckenberger:** Conceptualization, Resources, Writing – review & editing, Project administration, Funding acquisition. **Stephanie Tanadini-Lang:** Conceptualization, Resources, Writing – review & editing, Supervision, Project administration, Funding acquisition.

## Declaration of Competing Interest

The authors declare the following financial interests/personal relationships which may be considered as potential competing interests: Financial support was partially provided by ViewRay Inc. (MASPAC study), the Swiss National Science Foundation R'Equip Program (grant 326030_177080/1), and the Clinical Research Priority Programme “Artificial intelligence in Oncological Imaging” of the University of Zurich.
