# Supplementary material for: Synthetic computed tomography for low-field magnetic resonance-only radiotherapy in head-and-neck cancer using residual vision transformers
Source: Phys Imaging Radiat Oncol. 2023 Jul 8;27:100471. doi: 10.1016/j.phro.2023.100471 (PMC10366636; doi:10.1016/j.phro.2023.100471)
Supplement: Supplementary data 1 [file mmc1.docx]

# **SUPPLEMENTARY MATERIAL A**


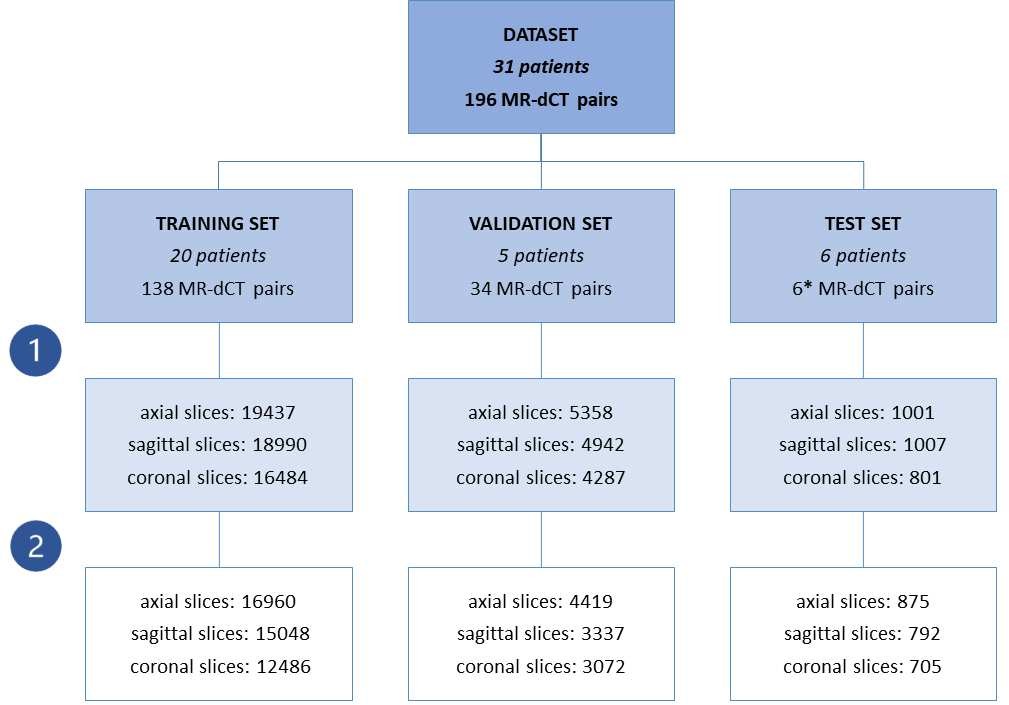


Fig. A. Flow diagram of the patient and 2D slice selection process. 1: Selection of slices that either contain the PTV or are situated less than 20 mm away in the craniocaudal, mediolateral, and anteroposterior directions for the axial, sagittal, and coronal sets, respectively. 2: Exclusion of slices with artefacts (i.e, slices with a maximal HU value above 2200 HU. *: only the ground-truth MR-Dct pair from the first day of treatment is used in the test set.

# **SUPPLEMENTARY MATERIAL B**

Table B. Patients characteristics.

|  | **Training** | **Validation** | **Test** |
| --- | --- | --- | --- |
|  |  |  |  |
| **Patients** | 20 | 5 | 6 |
|  |  |  |  |
| **Sex** |  |  |  |
| *Male* | 13 | 3 | 6 |
| *Female* | 7 | 2 | 0 |
|  |  |  |  |
| **Age** |  |  |  |
| *median* | 61 | 61 | 70 |
| *range* | [43 - 72] | [55 - 71] | [63 - 79] |
|  |  |  |  |
| **PTV size** |  |  |  |
| **[cm3]** |  |  |  |
| *median* | 488 | 484 | 669 |
| *range* | [236 - 700] | [338 - 715] | [443 - 914] |
|  |  |  |  |
| **Tumour Location** | |  |  |
| *Oropharynx* | 16 | 2 | 4 |
| *Larynx* | 3 | 1 | - |
| *Hypopharynx* | 1 | 2 | 2 |
| *Oral Cavity* | 1 | - | - |
|  |  |  |  |
| **Stage** |  |  |  |
| *I-II* | 3 | - | 2 |
| *III-IV* | 17 | 5 | 4 |

# **SUPPLEMENTARY MATERIAL C**


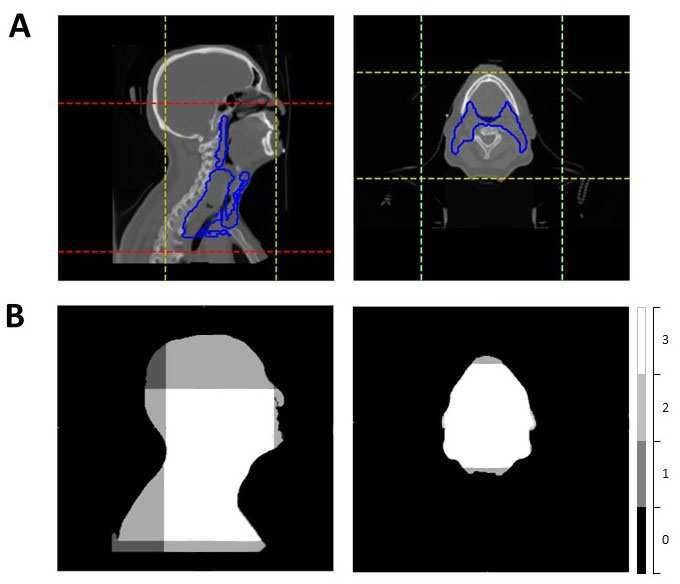


Fig. C. A: The axial, sagittal and coronal slices covered the whole PTV (blue) plus 15 mm margins in the axial (red), sagittal (green) and coronal (yellow) directions, respectively. B: A 3D body mask was built after the union of the corresponding axial, sagittal and coronal body masks.

# **SUPPLEMENTARY MATERIAL D**

- The loss function (L) to train ResViT:

$L = \lambda_{pix}L_{pix} + \lambda_{adv}L_{adv}$ (Eq. D1)

$L_{pix} = E \left[ \left\| sCT - dCT \right\| \right]$ (Eq. D2)

$L_{adv} = - E \left[ {D(concat(MR, dCT)}^{2} \right] - E \left[ {(D(concat(MR, sCT)-1)}^{2} \right]$ (Eq. D3)

where D is the discriminator.

- The mean absolute error (MAE):

$MAE = \frac{\sum_{1}^{n} \left| {CT}_{j} - {sCT}_{j} \right|}{n}$ (Eq. D4)

with n = number of voxels in the region of interest.

- The root mean squared error (RMSE):

$RMSE = \sqrt[2]{\frac{\sum_{1}^{n} \left( {CT}_{j} - {sCT}_{j} \right)^{2}}{n}}$ (Eq. D5)

with n = number of voxels in the region of interest.

- The peak-signal-to-noise-ratio (PSNR):

$PSNR = 10 \log\frac{{MAX(CT)}^{2}}{MSE}$ (Eq. D6)

- The structural similarity index measure (SSIM):

$SSIM = \frac{\left( 2 \mu_{sCT}\mu_{dCT}+ c_{1} \right)\left( 2 \sigma_{sCTdCT}+ c_{2} \right)}{\left( {\mu_{sCT}}^{2} + {\mu_{dCT}}^{2}+ c_{1} \right)\left( {\sigma_{sCT}}^{2} + {\sigma_{sCT}}^{2}+ c_{2} \right)}$ (Eq. D7)

where $c_{1}$ = ${(k_{1}L)}^{2}$, $c_{2}$ = ${(k_{2}L)}^{2}$, with $k_{1}$ = 0.01, $k_{2}$ = 0.03, L = dynamic range, µ = mean, σ = variance

- The Dice similarity coefficient (DSC):

$DSC = \frac{2 \left| X \cup Y \right|}{\left| X \right| + \left| Y \right|}$ (Eq. D8)

# **SUPPLEMENTARY MATERIAL E**

Table E. Results for the six test patients. MAE: mean absolute error, RMSE: root mean squared error, PSNR: peak-signal-to-noise ratio, SSIM: structural similarity index measure, GPR: gamma passing rates, DVH: dose volume histogram, GTV: gross tumour volume, PTV: planning target volume, D_mean_: mean dose, D_2_: near maximum dose, D_95_: coverage dose.

|  | | **Patient 1** | **Patient 2** | **Patient 3** | **Patient 4** | **Patient 5** | **Patient 6** |
| --- | --- | --- | --- | --- | --- | --- | --- |
| ***MAE [HU]*** | | 74 | 58 | 65 | 56 | 42 | 37 |
| ***RMSE [HU]*** | | 149 | 118 | 135 | 116 | 85 | 75 |
| ***PSNR*** | | 0.97 | 0.97 | 0.98 | 0.97 | 0.97 | 0.98 |
| ***SSIM*** | | 28.8 | 28.8 | 30.8 | 29.6 | 30.9 | 33.7 |
| ***GPR 2%/2mm*** | *50%* | 95.9 | 99.2 | 97.5 | 97.9 | 97.3 | 96.9 |
|  | *90%* | 92.1 | 98.4 | 94.0 | 94.9 | 95.2 | 92.7 |
| ***GPR 3%/3mm*** | *50%* | 99.5 | 100.0 | 99.8 | 99.8 | 99.8 | 99.7 |
|  | *90%* | 99.2 | 100.0 | 99.3 | 99.6 | 99.6 | 99.2 |
| ***Relative signed DVH deviations %*** | |  |  |  |  |  |  |
| *GTV_D_mean_* | | 0.39 | 0.57 | -0.25 | 0.96 | 0.78 | -0.39 |
| *Mandible_D_2_* | | 0.41 | 0.75 | -0.08 | 0.77 | 0.88 | -0.67 |
| *Oral Cav_D_mean_* | | 0.89 | 0.56 | -0.26 | -0.64 | 0.75 | -0.88 |
| *PTV1_D_2_* | | 0.43 | -0.96 | -0.06 | -0.21 | 0.55 | 0.79 |
| *PTV1_D_95_* | | 0.61 | 0.08 | -0.15 | 0.00 | 0.61 | 0.00 |
| *PTV1_D_mean_* | | 0.54 | 0.64 | -0.04 | -0.04 | 0.66 | 0.48 |
| *Parotid L_D_mean_* | | - | 0.06 | 0.42 | -0.66 | 0.46 | -0.65 |
| *Parotid R_D_mean_* | | 0.83 | -0.06 | -0.19 | -0.41 | 0.36 | -0.3 |
| *Salivary L_D_mean_* | | - | -0.08 | 0.4 | 0.25 | 0.58 | - |
| *Salivary R_D_mean_* | | - | 0.32 | -0.75 | 0.44 | 0.67 | - |
| *Spinal Cord_D_2_* | | -0.71 | 0.89 | -0.27 | 0.82 | 0.43 | 0.14 |

# **SUPPLEMENTARY MATERIAL F**


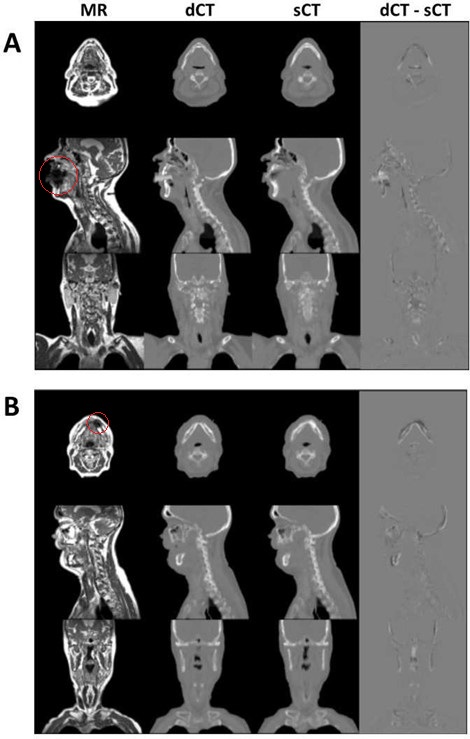


Fig. F. Axial, sagittal, and coronal slices of two test cases. A: The images of this patient present artefacts as a result of the mouth bite immobilisation technique. B: The images of this patient present a common dental artefact, which is corrected by density override in the dCT, but present in the sCT.

# **SUPPLEMENTARY MATERIAL G**


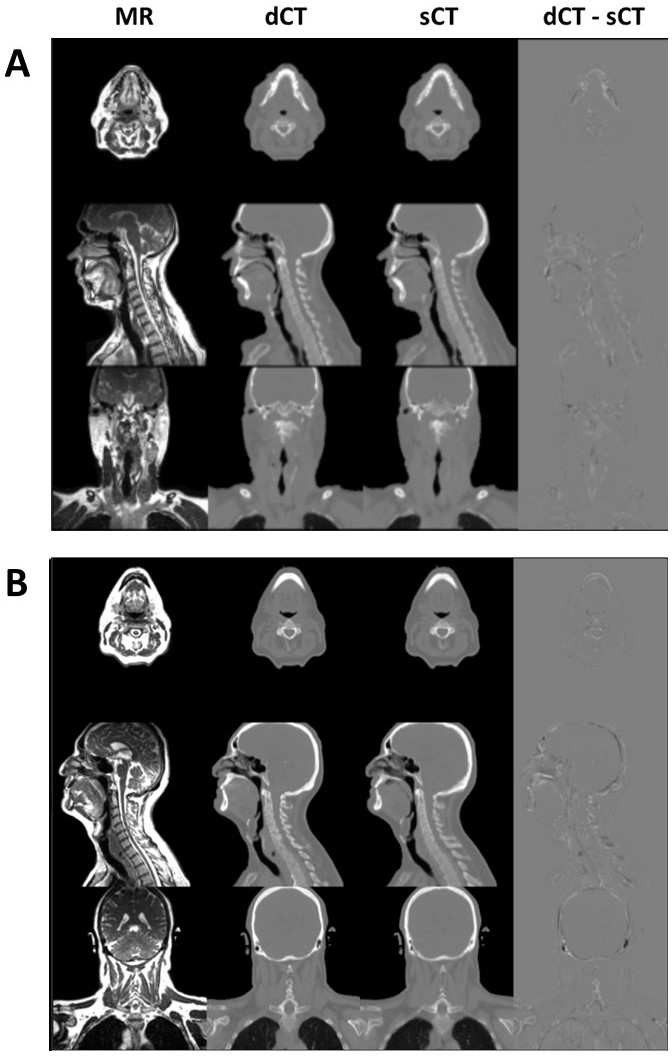


Fig. G. Axial, sagittal, and coronal slices of two test cases.

# **SUPPLEMENTARY MATERIAL H**


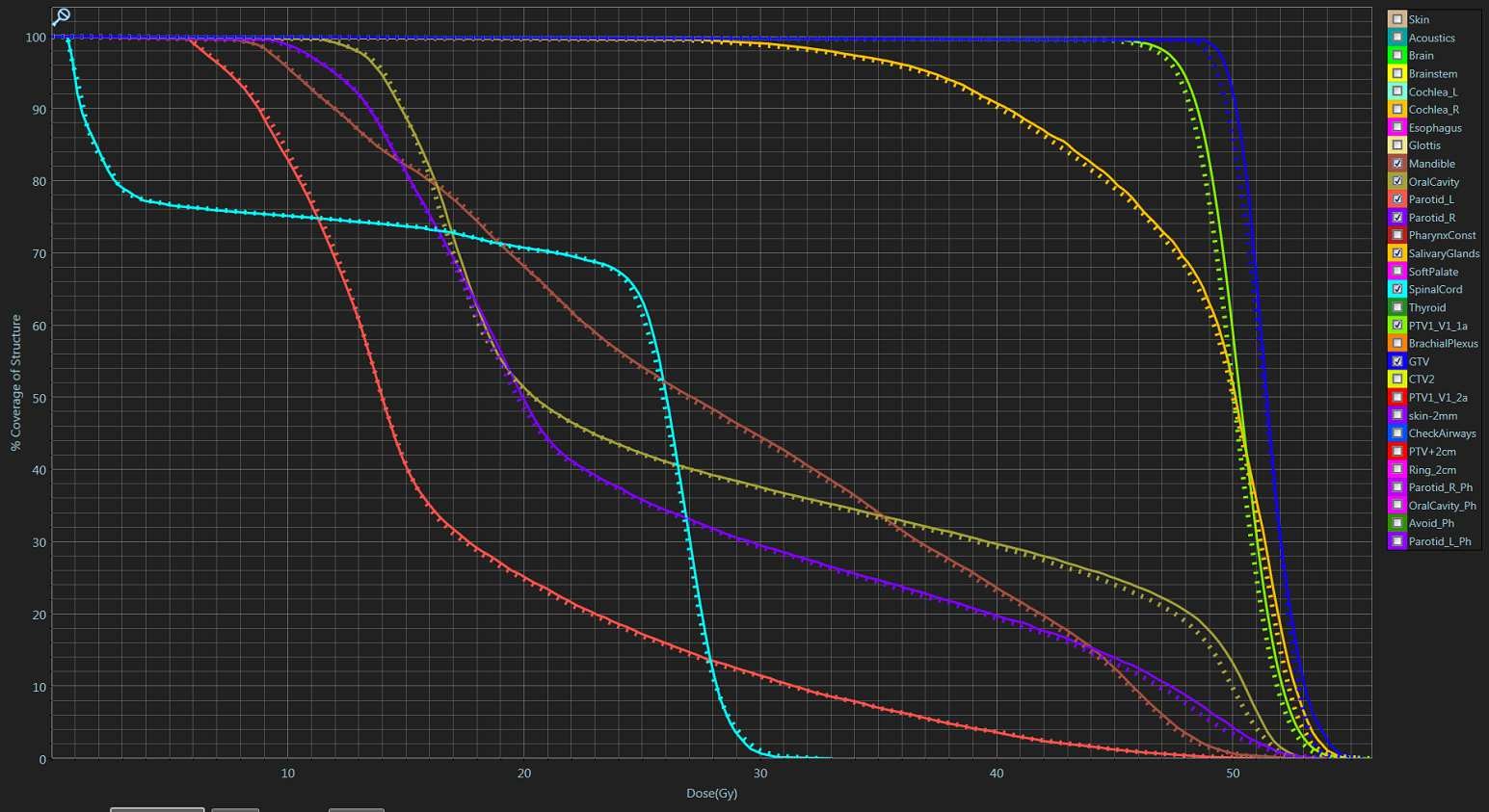


Fig. H.1. DVH of test patient 1 comparing dCT-based (dashed) and sCT-based (solid) dose distributions for the GTV, PTV, spinal cord, mandible, oral cavity, parotid glands, and submandibular glands.


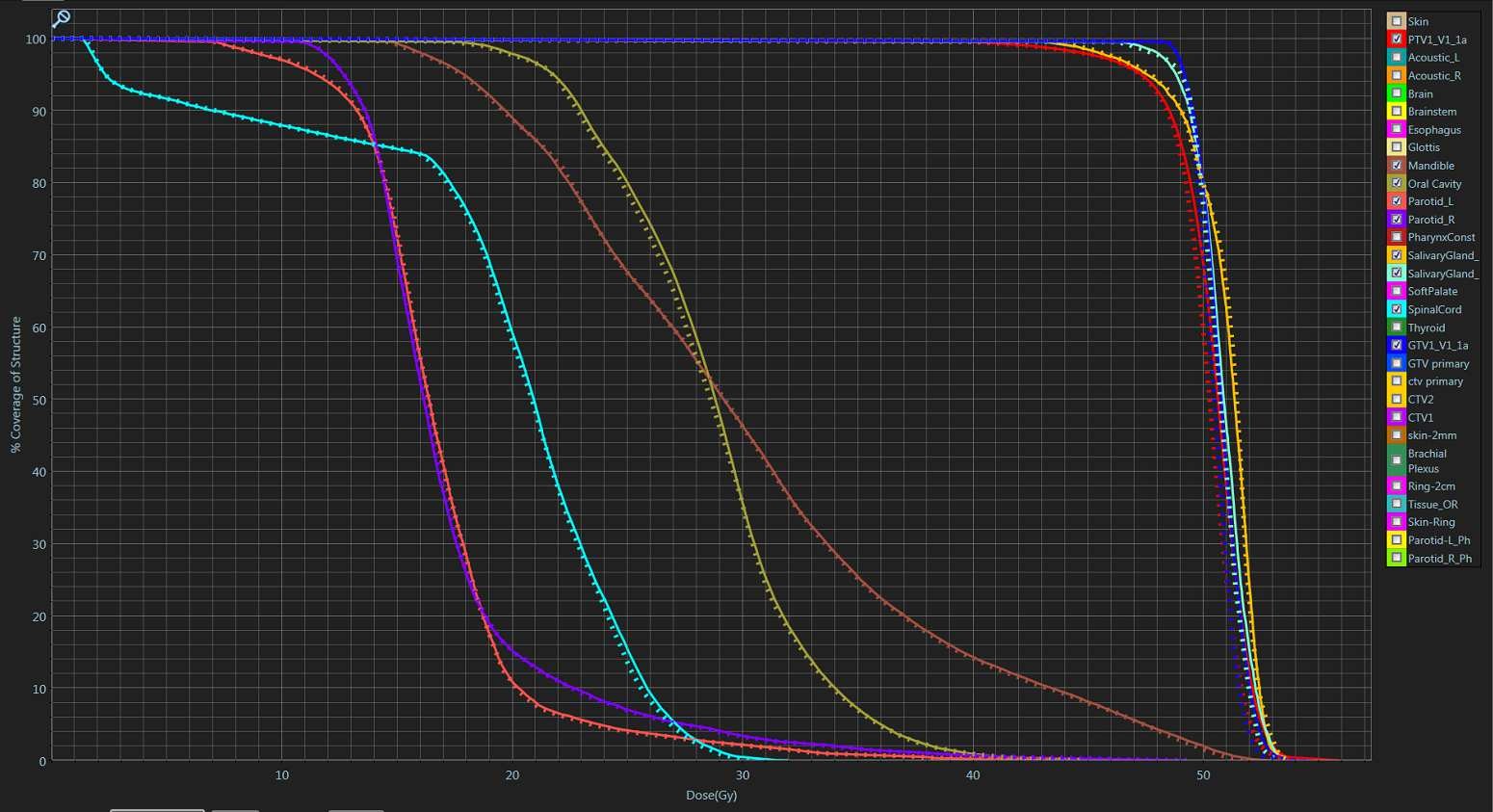


Fig. H.2. DVH of test patient 2 comparing dCT-based (dashed) and sCT-based (solid) dose distributions for the GTV, PTV, spinal cord, mandible, oral cavity, parotid glands, and submandibular glands.


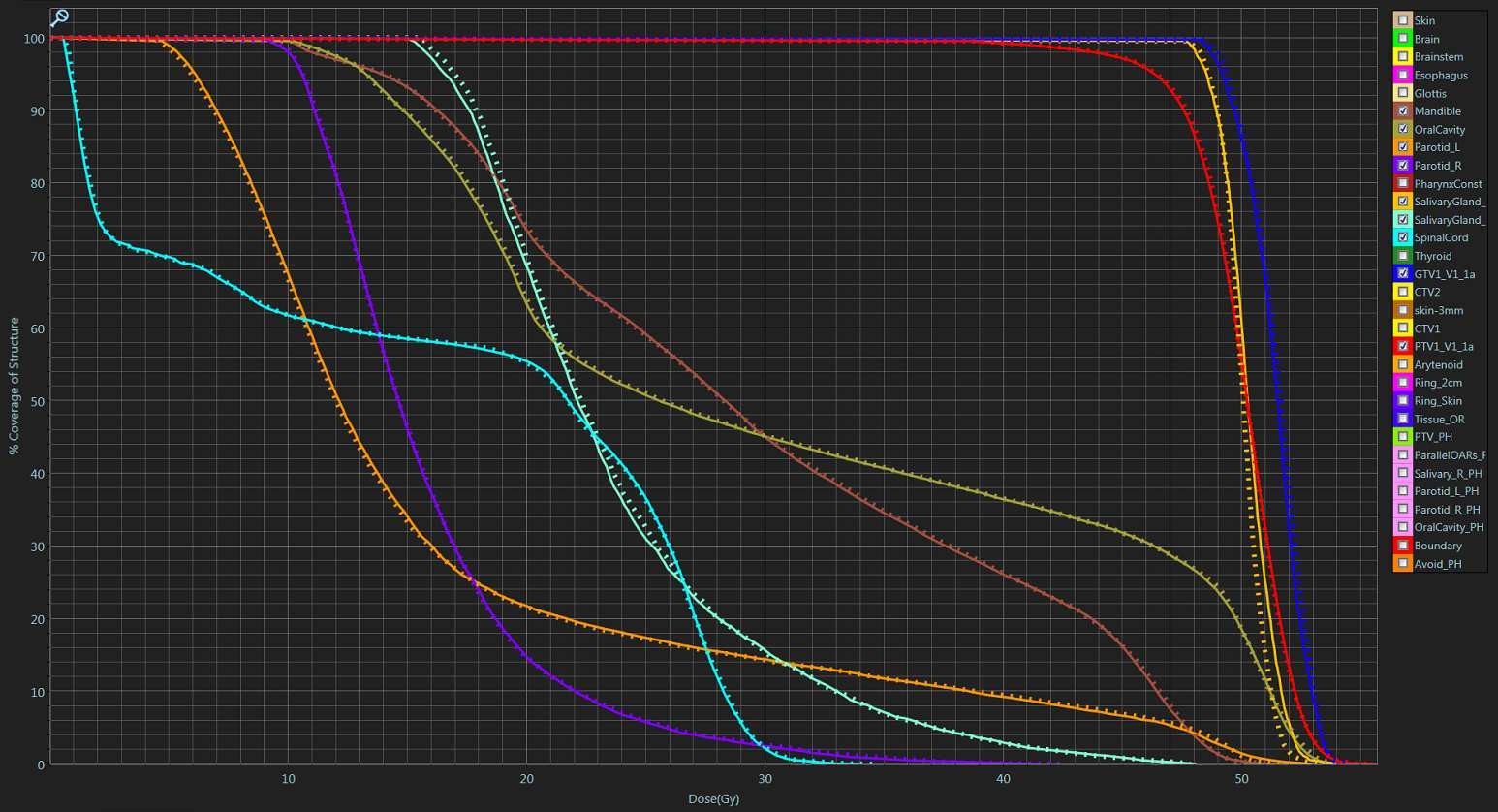


Fig. H.3. DVH of test patient 3 comparing dCT-based (dashed) and sCT-based (solid) dose distributions for the GTV, PTV, spinal cord, mandible, oral cavity,parotid glands, and submandibular glands.


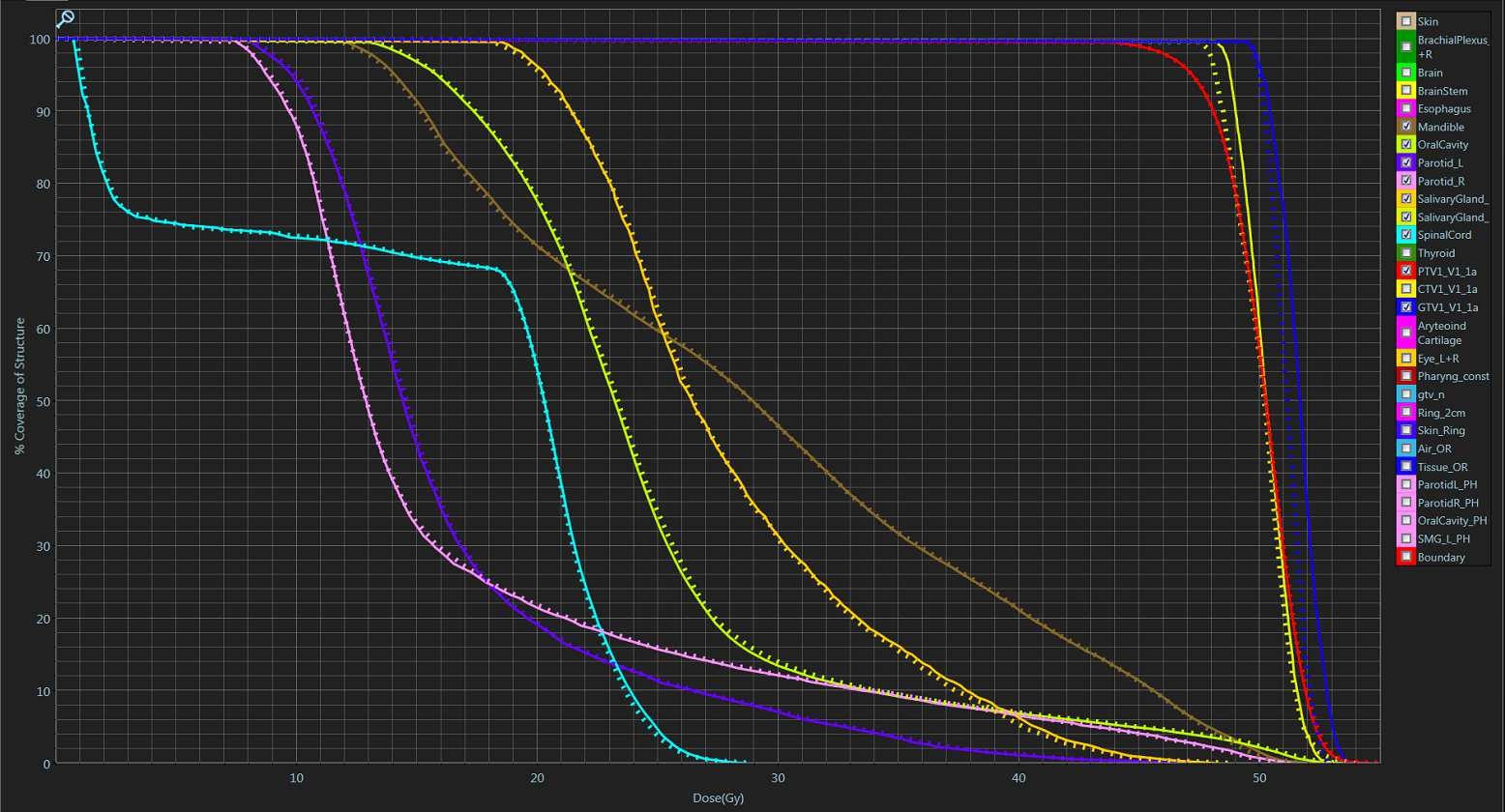


Fig. H.4. DVH of test patient 4 comparing dCT-based (dashed) and sCT-based (solid) dose distributions for the GTV, PTV, spinal cord, mandible, oral cavity, parotid glands, and submandibular glands.


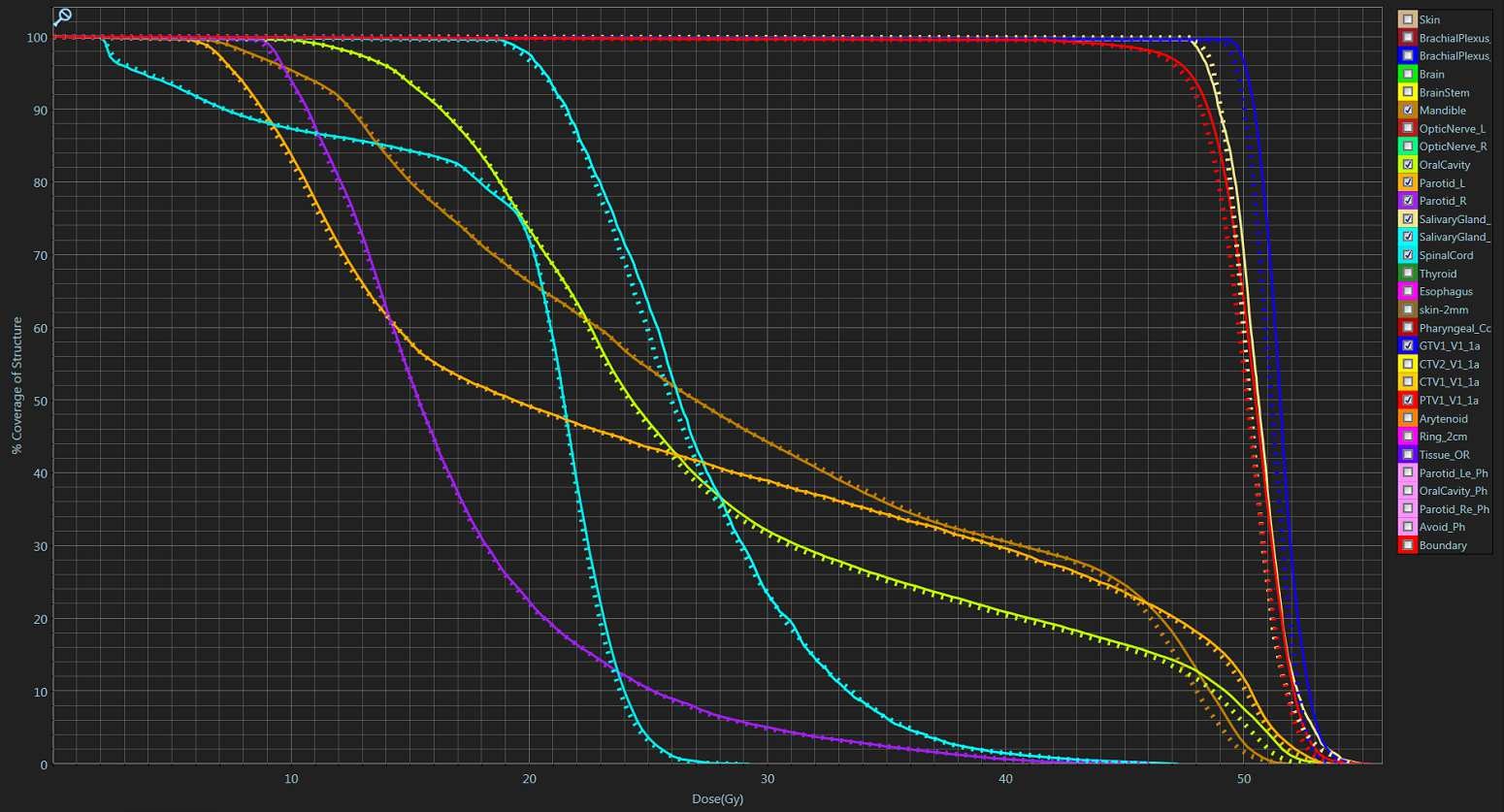


Fig. H.5. DVH of test patient 5 comparing dCT-based (dashed) and sCT-based (solid) dose distributions for the GTV, PTV, spinal cord, mandible, oral cavity, parotid glands, and submandibular glands.


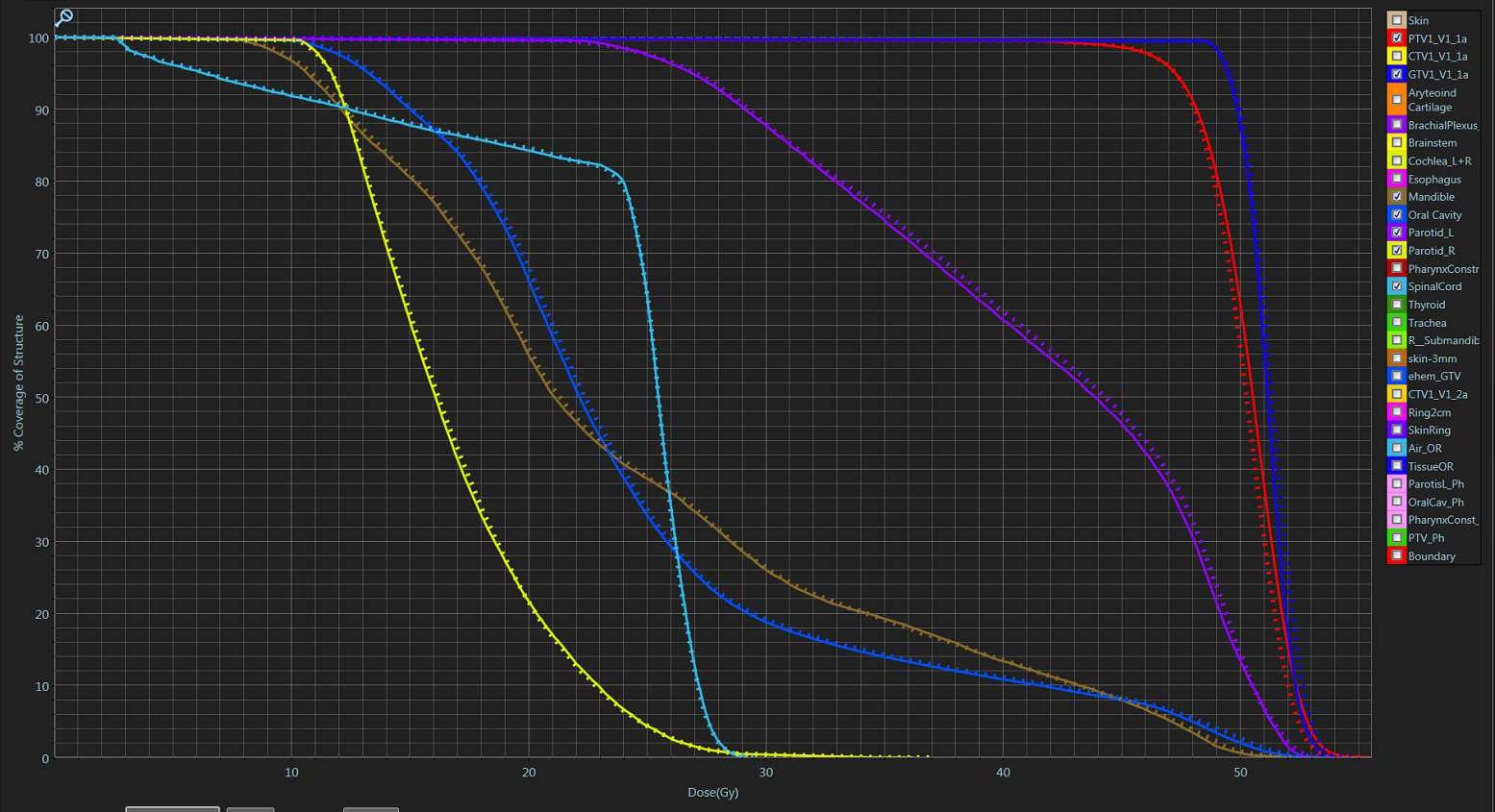


Fig.H.6. DVH of test patient 6 comparing dCT-based (dashed) and sCT-based (solid) dose distributions for the GTV, PTV, spinal cord, mandible, oral cavity, parotid glands, and submandibular glands.

# SUPPLEMENTARY MATERIAL I

- The Hausdorff Distance (HD): $HD (X,Y) =max \{\max_{x \epsilon X} \min_{y \epsilon Y} d(x,y), \max_{y \epsilon Y} \min_{x \epsilon X} d(x,y))$(Eq. I1)


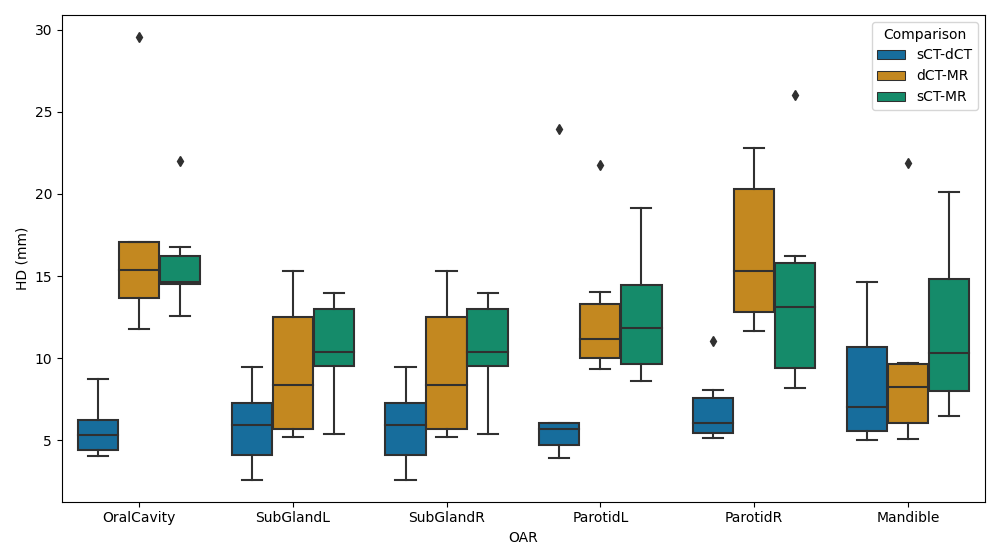


Fig. I.1. Boxplot of the calculated HDs between the automatically generated contours on the sCT and the automatically generated contours on the dCT (blue); between the automatically generated contours on the dCT and the manual contours on the MR (orange); and between the automatically generated contours on the sCT and the manual contours on the MR (green).

- The Jaccard Index: $Jaccard = \frac{\left| X \cup Y \right|}{\left| X \cap Y \right|}$ (Eq. I2)


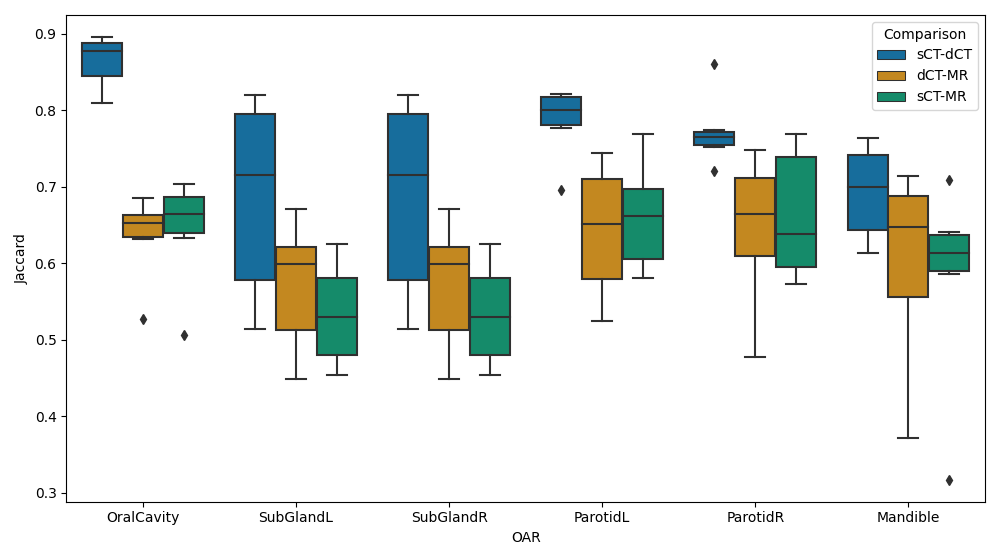


Fig. I.2. Boxplot of the calculated Jaccard indices between the automatically generated contours on the sCT and the automatically generated contours on the dCT (blue); between the automatically generated contours on the dCT and the manual contours on the MR (orange); and between the automatically generated contours on the sCT and the manual contours on the MR (green).

- The Mean Distance to Agreement (MDA): $MDA (X,Y) = \frac{\sum_{1}^{n} \left| d(x,y) \right|}{n}$ (Eq. I3),

where n are pairs of nearest neighbor points.

Fig. I.3. Boxplot of the calculated MDAs between the automatically generated contours on the sCT and the automatically generated contours on the dCT (blue); between the automatically generated contours on the dCT and the manual contours on the MR (orange); and between the automatically generated contours on the sCT and the manual contours on the MR (green).

# **SUPPLEMENTARY MATERIAL J**

Table J. Comparison with previous studies investigating DL-based sCT generation for MR-only radiotherapy in HN cancer and with previous studies investigating DL-based sCT generation from low-field MR images for other cancer sites.

|  | **Magnetic field [T]** | **MR**  **contrast** | **Number**  **patients** | **MAE [HU]**  **[mean ± std]** | **DVH** | **Gamma [%]**  **2%/2mm**  **[mean ± std]** |
| --- | --- | --- | --- | --- | --- | --- |
| ***Other HN studies*** |  |  |  |  |  |  |
| *Dinkla et al.*[31] | 3 | T2+Dixon | 34 | 75 ± 9 |  | 95.6 ± 2. 9 |
| *Klages et al.*[35] | 3 | T1+Dixon | 20 | 92 ± 14 | < 2 % |  |
| *Largent et al.*[30] | 1.5 | T2 | 8 | 83 ± 49 |  |  |
| *Palmer et al.*[29] | 1.5 | T1+Dixon | 44 | 67 ± 14 | < 2 % | 99.4 [95.7 – 99.9] |
| *Peng et al.*[36] | 3 | T1 | 173 | 70 ± 9 | < 2 % | 98.7 ± 2. 9 |
| *Qi et al.*[38] | 3 | T1, T2, T1+contrast, T1+contrast+Dixon | 45 | 70 ± 12 |  | 97.6 ± 1.3 |
| *Tie et al.* [32] | 1.5 | T1, T1+contrast, T2 | 32 | 76 ± 15 |  |  |
| *Thummerer et al.* [37] | 3 | T1 spoiled gradient echo | 27 | 65 ± 4 | < 6 % | 93.5 ± 3.4 |
| *Wang et al.*[33] | 1.5 | T2 | 33 | 131 ± 24 |  |  |
| ***Other low-field studies*** |  |  |  |  |  |  |
| *Cusumano et al.* [40] | 0.35 | T1/T2* | 120 | 79 ± 19  54 ± 12 | < 2.5 % | 98.7 ± 1.1  99.0 ± 0.7 |
| *Fu et al.* [45] | 0.35 | T1/T2* | 12 | 90 ± 19 | < 1 % | 98.7 ± 1.5 |
| *García-Hernández et al.* [44] | 0.35 | T1/T2* | 76 | 26 ± 13 | < 4 % | 91.6 ± 6.0 |
| *Hsu et al.* [43] | 0.35 | T1/T2* | 57 | 30 ± 4 | < 2.5 % | 99.9 ± 0.1 |
| *Lapaeva et al.* [39] | 0.35 | T1/T2 | 168 | 70 ± 20 | < 2 % | 99. 5 ± 0.6 |
| *Lenkowicz et al.* [41] | 0.35 | T1/T2* | 60 | 55 ± 11 | < 5 % | 95.5 ± 5.9 |
| *Zimmermann et al.* [42] | 0.35 | T2 | 12 | 44 ± 3 | < 2 % | 98 |
| ***Our approach*** | **0.35** | **T1/T2** | **31** | **56 ± 13** | **< 1 %** | **97.4 ± 1.0** |
